# Application of high-resolution genomic profiling in the differential diagnosis of liposarcoma

**DOI:** 10.1186/s13039-017-0309-5

**Published:** 2017-03-16

**Authors:** Magdalena Koczkowska, Beata Stefania Lipska-Ziętkiewicz, Mariola Iliszko, Janusz Ryś, Markku Miettinen, Jerzy Lasota, Wojciech Biernat, Agnieszka Harazin-Lechowska, Anna Kruczak, Janusz Limon

**Affiliations:** 1 Department of Biology and Genetics, Medical University of Gdansk, 1 Debinki Street, 80-211 Gdansk, Poland; 2 Department of Tumor Pathology, M. Sklodowska-Curie Memorial Institute of Oncology, 11 Garncarska Street, 31-115 Krakow, Poland; 3 Laboratory of Pathology, National Cancer Institute, Building 10, Room B1B47, 10 Center Drive, Bethesda, 20892 MD USA; 4 Department of Pathology, Medical University of Gdansk, 17 Smoluchowskiego Street, 80-214 Gdansk, Poland

**Keywords:** Liposarcoma, Array-based comparative genomic hybridization (array-CGH), Copy number aberrations, Genomic profiling, Genomic imbalances

## Abstract

**Background:**

Rarity and heterogeneity of liposarcomas (LPS) make their diagnosis difficult even for sarcoma-experts pathologists. The molecular mechanism underlying the development and progression of liposarcomas (LPS) remains only partially known. In order to identify and compare the genomic profiles, we analyzed array-based comparative genomic hybridization (array-CGH) profiles of 66 liposarcomas, including well-differentiated (WDLPS), dedifferentiated (DDLPS) and myxoid (MLPS) subtypes.

**Results:**

Copy number aberrations (CNAs) were identified in 98% of WDLPS and DDLPS and in 95% of MLPS cases. The minimal common region of amplification at 12q14.1q21.1 was observed in 96% of WDLPS and DDLPS cases. Four regions of CNAs, including losses of chromosome 6, 11 and 13 and gains of chromosome 14 were classified as recurrent in DDLPS; at least one was identified in 74% of DDLPS tumors. The DDLPS-associated losses were much more common in tumors with increased genomic complexity. In MLPS, the most frequent CNAs were losses of chromosome 6 (40%) and gains of chromosome 1 (30%), with the minimal overlapping regions 6q14.1q22.31 and 1q25.1q32.2, respectively.

**Conclusions:**

Our findings show that the application of array-CGH allows to delineate clearly the genomic profiles of WDLPS, DDLPS and MLPS that reflect biological differences between these tumors. Although CNAs varied widely, the subtypes of tumors have characteristic genomic profiles that could facilitate the differential diagnosis of LPS subtypes, especially between WDLPS and DDLPS.

**Electronic supplementary material:**

The online version of this article (doi:10.1186/s13039-017-0309-5) contains supplementary material, which is available to authorized users.

## Background

Liposarcomas (LPS), the most common soft tissue sarcomas accounting for less than 1% of all human cancer cases, display remarkable clinical and pathological heterogeneity. Morphologically, liposarcomas are divided into four main subtypes: well-differentiated (WDLPS), dedifferentiated (DDLPS), myxoid/round cell (MLPS/MRLPS) and pleomorphic liposarcomas (PLPS). WDLPS represents 40–50% of LPS, followed by MLPS (30–35%) [1, 2].

Somatic copy number aberrations (CNAs) occur commonly in human cancer and evaluation of their characteristic patterns may be used as a diagnostic tool, especially in soft tissue sarcomas [3]. Knowledge of alterations in genome structure could also facilitate identification of corresponding oncogenes or tumor suppressor genes associated with the pathogenesis or progression of the disease. So far, CNAs in LPS were mostly evaluated using classical cytogenetic and targeted FISH approaches. It allowed identification of supernumerary ring giant chromosomes and double-minute chromosomes (dmin) in WDLPS and DDLS. These chromosomes contain amplified segments from the 12q13q15 region, including *MDM2, CDK4* and *HMGA2* oncogenes. Consequently, evaluation of 12q13q15 amplification has been applied clinically as it allows for distinguishing WDLPS/DDLPS from benign adipocytic tumors [4, 5]. However, the differential diagnosis between WDLPS and DDLPS is much more challenging, because about 10% of DDLPS are the recurrences of WDLPS as a non-lipogenic sarcoma of variable histological grade. Little is known about the molecular mechanism of dedifferentiation and no genetic alteration has been identified as contributing to this process yet. Recently, it has been suggested that the number of dedifferentiation events in LPS could be underestimated and that actually DDLPS might be the most common histological subtype [6]. MLPS, the third subtype of LPS, is distinguished by the presence of a specific translocation t(12;16) [7] or t(12;22) [8] that is the key genetic aberration, extremely helpful in differential diagnosis between MLPS and myxofibrosarcomas [5]. These unique chromosomal translocations, detected in more than 95% of cases, result from a fusion of the segments of the *DDIT3* gene (12q13) and the *FUS* gene (16p11) or the *EWSR1* gene (22q12) [1, 9].

In recent years, the application of high-resolution methods, such as array-CGH, results in a significant progress in the whole-genome analysis. A few studies published so far have evaluated application of array-CGH and/or whole exome sequencing (WES) techniques in the cohort of patients with LPS [9–11]. Tap et al. (2011) have reported gains in 1p32 with *JUN* amplification and 6q23 as frequent areas of interest in WDLPS and DDLPS, whereas the loss of 19q13 is thought to be associated with the poorer prognosis [10]. Up to now, no detailed genomic profiles of MLPS using high - resolution array-CGH method have been published.

In this study, we performed array-based comparative genomic hybridization (array-CGH) analyses on 69 LPS tumors aimed at identification of specific patterns of chromosomal aberrations that reflect biological differences between these tumors. Accordingly, the diagnostic value of combining morphology with genetic testing was estimated in the group of patients with LPS.

## Methods

### Tumor specimens

In total, 69 fresh-frozen tissue samples from 53 patients diagnosed with liposarcomas were included in this study: 23 WDLPS (from 18 patients), 23 DDLPS (from 16 patients) and 23 MLPS (from 19 patients). All tissue samples have been stored in the archives of the Department of Biology and Genetics, Medical University of Gdansk. The histological subtypes and tumor tissue content of each sample were evaluated independently by two sarcoma-expert pathologists. The clinicopathological data (patients’ gender and age, tumor type and its site of development) are presented in Additional file 1: Table S1. Briefly, the studied group consisted of 28 primary tumors, 31 local recurrences and ten metastases. The median age at initial diagnosis was 50 years (range 32–82). The retroperitoneum (39%, 27/69) was the most prevalent location followed by the extremities (33%, 23/69).

### DNA extraction

Genomic DNA, after verification of neoplastic cell content as exceeding 70%, was extracted from a fresh-frozen tumor tissue sample according to salting-out protocol [12]. Array-CGH analyses were performed on archival material. In most cases peripheral blood from patients was not available, therefore a pool of female DNA, isolated by using QIAamp DNA Blood Midi Kit (Qiagen, Hilden, Germany), was used as the reference DNA.

### Array-CGH analysis

Array-CGH was performed at resolution of 10 kbp using Human CGH 2.1M Whole-Genome Tilling Array (NimbleGen, Roche, Basel, Switzerland) following the instructions provided by manufacturer with modification as previously described [13]. Arrays were scanned at 2 μm with MS200 Microarray Scanner (NimbleGen, Roche, Basel, Switzerland) and analyzed with Deva v1.0.2 and Nexus Copy Number 7.5 softwares (NimbleGen, Roche, Basel, Switzerland and BioDiscovery, El Segundo, CA, USA, respectively). Extracted arrays with a DRL spread <0.3 were included in the analysis (average DRLs = 0.15). A minimum of five consecutive probes were required to define a region as a CNA. All identified genomic imbalances were verified in the in-house database, containing >1000 benign copy number variations (CNVs), identified in local populations as well as in online database of genomic variants (DGV; http://dgv.tcag.ca) [14]. Numbering of map positions was based on hg18 (NCBI36 reference sequence).

### Karyotyping and FISH

Cytogenetic studies were performed using conventional GTG-banding of tumor cells metaphase chromosomes at a 550 band level following standard protocol after the digestion with collagenase [15]. In each analysis from 5 to 30 metaphases were evaluated. In MLPS cases where chromosomal translocation t(12;16) or t(12;22) was not noted by karyotype studies, FISH using Vysis LSI DDIT3 (CHOP) Dual Color, Break Apart Rearrangement Probe (Abbott Molecular Inc, Des Plaines, IL, USA) was performed.

### Quantitative real-time PCR

Small deletions (<300 kbp) were validated by quantitative real-time PCR (qPCR) performed on Light Cycler 480 System (Roche, Basel, Switzerland) using specific FAM pre-labelled probes from Universal Probe Library (Roche, Basel, Switzerland). Target genes within the deleted or duplicated regions were assessed against a control sequence at Xq28 and two reference genes: *GPR15* (3q11.2) and *ERMP1* (9p24.1). All samples were run in triplicates. The dosage of target genes relative to reference genes normalized to control DNA was assessed.

### Statistical analysis

For univariate analysis, Fisher’s exact test and Mann-Whitney test or Kruskal-Wallis test were used to compare categorical and continuous variables, respectively. Analyses were performed with the STATISTICA 10 software (StatSoft Inc, Tulsa, OK, USA).

## Results

### General overview

Normal karyotype established by classical karyotyping was observed in 11.6% (8/69) of tumors. Ninety-six percent (66/69) of tumors were successfully profiled by array-CGH. The remaining three samples failed the analysis because of high degree of DNA degradation and were excluded from further analyses. The detailed list of all aberrations detected in each tumor by classic and molecular cytogenetics is presented in Additional file 2: Table S2.

**Fig. 1:**
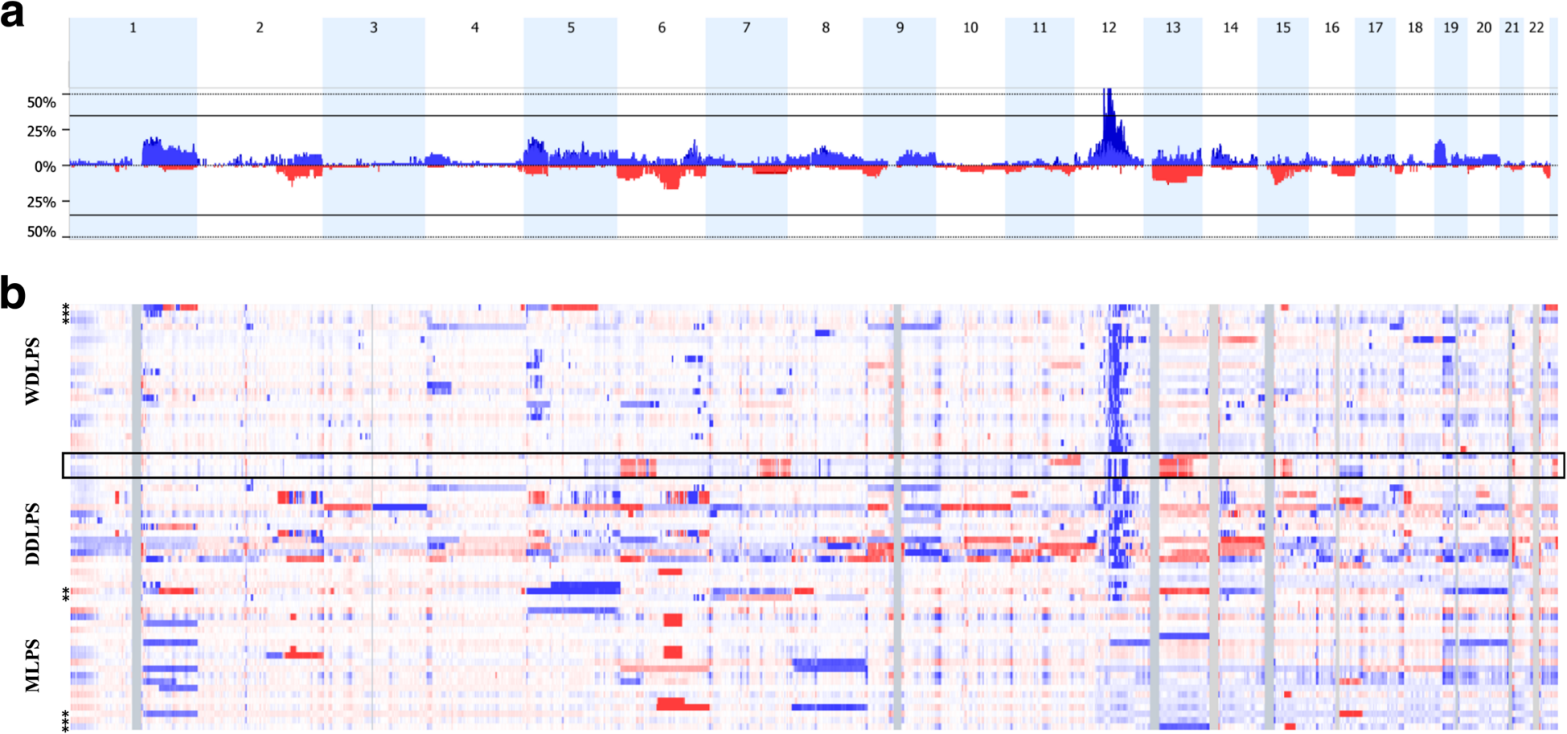
Copy number aberrations (CNAs) identified by array-CGH in 66 LPS samples. **a** Frequency plot of CNAs in all LPS samples. **b** Heat map of the CNAs of 66 LPS tumor samples grouped by histological subtypes. *Red* and *blue bars* depict percentage of tumors with losses and gains, respectively in the corresponding region of chromosome. X-axis shows the consecutive chromosome numbers. Color intensity on heat map corresponds to the normalized fluorescence log2 ratio from array-CGH experiments. *Asterisks* (*) indicate samples with the normal genome profile established by conventional karyotyping. Samples denoted by the *black* frame represent tumors with diagnosis refinement based on array-CGH results

Genomic imbalances were revealed in 97% of the tumors (Fig. 1), including 98% (45/46) of WDLPS and DDLPS and 95% (19/20) of MLPS. CNAs affected on average 9.8% of the genome in DDLPS (range: 0.4–29.2%) vs. 1.9% (range: 0–10.3%) and 3.8% (range: 0–13.3%) in WDLPS and MLPS, respectively; the difference being statistically significant (*p* < 0.001; Kruskal-Wallis test). The losses were much more common in DDLPS than in WDLPS (*p* < 0.001 OR = 0.02 95% Cl [0.002–0.146]), while no statistically significant difference in number of gains was observed. No amplification region was observed in MLPS. The comparison of all tumors, irrespective of the histological subtype, demonstrated that losses of chromosome 6 and gains of chromosome 5 were found more common in recurrences and metastases than in the primary tumors (*p* = 0.009 and *p* = 0.042, respectively). Besides, the most complex genomic profiles were observed in recurrences (6.8%; range: 0.1–29.2%), followed by metastases and primary tumors (5.3%; range: 0.7–11.9% and 3.5%; range: 0–24.1%, respectively).

### WDLPS/DDLPS

**Fig. 2:**
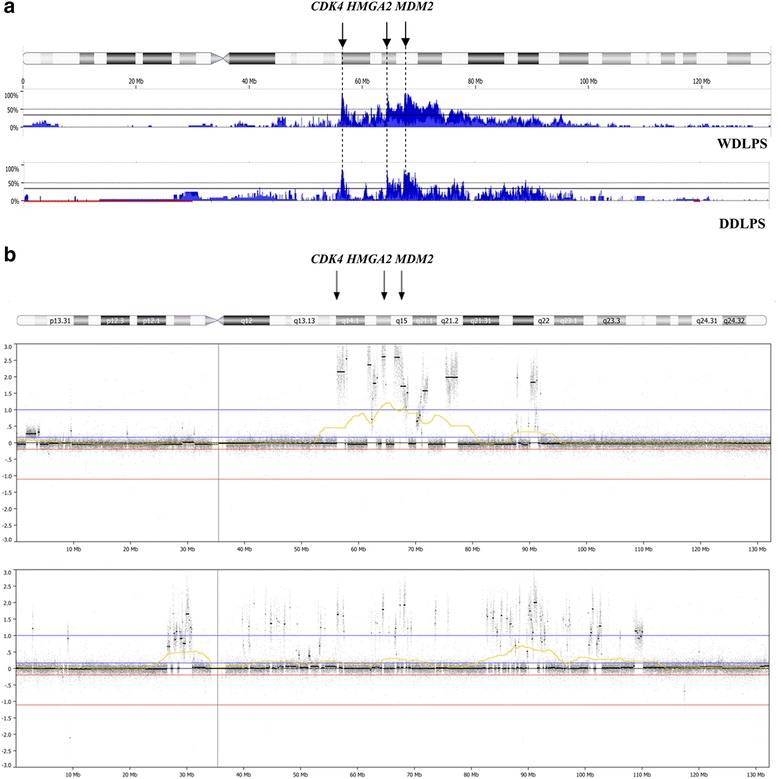
Array-CGH profile of chromosome 12 in WDLPS and DDLPS tumors. **a** Penetrance plots of copy number aberrations (CNAs) in 46 cases of WDLPS and DDLPS. **b** Examples of genomic imbalances on chromosome 12 detected by array-CGH. *Blue bars* indicate the percentage of tumors with an amplification in the corresponding region of the chromosome 12. *Black arrows* depict three peaks of amplification in this region with *CDK4, HMGA2* and *MDM2* loci. Increased resolution of array-CGH technique allowed for the establishing the amplification level of known oncogenes associated with WD/DDLPS pathogenesis

The most frequently affected chromosomal region in WDLPS and DDLPS was 12q, gained in 44/46 of both LPS subtypes, with the minimal common region at 12q14.1q21.1. The three peaks of amplification in this region were localized in the vicinity of *CDK4, HMGA2* and *MDM2* loci (Fig. 2). These genes were amplified in 95.7, 91.3, 95.7% of WDLPS and 91.3, 87, 91.3% of DDLPS, respectively.

**Fig. 3:**
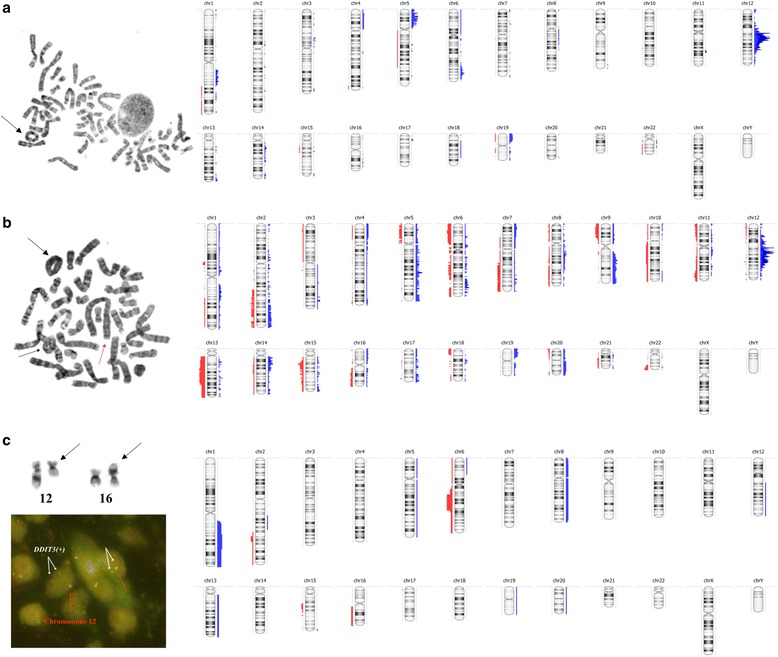
Comparison of the standard diagnostic approach and array-CGH in WDLPS, DDLPS and MLPS tumors. On the left the conventional karyotyping and/or FISH results are shown. *Black arrows* depict the supernumerary ring chromosomes in WDLPS and DDLPS tumors (**a**–**b**) and the balanced translocation t(12;16) in MLPS sample (**c**). *DDIT3* break apart probe was used in FISH analysis (one *orange* and one *green* signal pattern indicate a rearrangement of the *DDIT3* gene region). On the right the overview of all copy number aberrations (CNAs) detected by array-CGH is presented. *Red* and *blue* colors represent losses and gains, respectively

Besides, the commonly involved chromosomal regions in WDLPS were gains of short arms of chromosome 5 (9/23; 39%) and 19 (6/23; 26%) (Fig. 3a). The gain of chromosome 19p with the minimal overlapping region of 18 Mbp at 19p13.3p13.11, encompassing a total number of 586 genes, was more often detected in tumors with increased genomic complexity (*p* = 0.019; Mann – Whitney test). In addition, a homozygous deletion of 155 kbp at 8p11.23p11.22, encompassing *ADAM3a* and *ADAM5* genes, was found in 10 of 23 (44%) WDLPS tumors. Quantitative PCR analysis of matched tumor and normal tissue samples revealed constitutional and not somatic character of this aberration. The overall frequency of this CNV in in-house population-matched database has been estimated at 6.5%.

DDLPS were characterized by more numerous aberrations than WDLPS, the most common being losses of chromosomes 13 and 15 (Fig. 3b). The deletions of the long arms of chromosome 15 were found exclusively in the local recurrences (6/23; 26%), while aberrations of chromosome 13, identified in 30% of DDLPS (7/23), were characteristic for tumors located in the abdomen (*p* = 0.058 and *p* = 0.002, respectively). The minimal overlapping region of 65 Mb at 13q11q31.1 covered a total number of 382 genes, among which nine genes are known to be associated with cancer pathogenesis (*ZMYM2, CDX2, FLT3, BRCA2, LHFP, TTL, FOXO1, LCP1, RB1*). In addition, the losses of chromosome 11 and 13, found exclusively in DDLPS, were more frequently observed in the DDLPS tumors with increased genomic complexity (*p* = 0.027 and *p* = 0.049, respectively; Mann – Whitney test).

Genomic aberrations present considerably more (i.e. by at least 30%) often in DDLPS than in WDLPS have been classified as DDLPS-associated (Table 1). These included: losses of chromosome 6, 11 and 13q and gains of chromosome 14q. At least one of the DDLPS-associated CNA was identified in 74% of DDLPS tumors (17/23); while two such CNAs were present in 39% (9/23) and three or more in 13% (3/23) of DDPLS.

**Table 1:** Copy number aberrations associated with DDLPS subtype

Copy number aberration	WDLPS *n* (%)	DDLPS *n* (%)	*P* value (2-tailed Fisher’s exact test)
**Losses of chromosome 6**	**0**	**9 (39)**	**0.002**
6p23p22.2 6q11.1q12 6q15q22.33	0 0 0	6 (26) 3 (13) 3 (13)	**0.022** 0.233 0.233
**Losses of chromosome 11** ^**a**^	**0**	**6 (26)**	**0.022**
11p15.5p13 11q23.2q24.2	0 0	3 (13) 4 (17)	0.233 0.109
**Losses of chromosome 13q** ^**a**^	**0**	**7 (30)**	**0.009**
13q11q31.1	0	7 (30)	**0.009**
**Gains of chromosome 14q**	**3 (13)**	**10 (43)**	**0.047** OR = 0.2 95% Cl [0.05–0.85]
14q21.2q21.3	2 (9)	9 (39)	**0.035**
			OR = 0.2 95% Cl [0.03–0.79]
14q32.2q32.31	2 (9)	2 (9)	1

^a^CNAs associated with increased genomic complexity of DDLPS tumors

### MLPS

The presence of the specific genomic translocation t(12;16) was identified in 18 of 20 MLPS tumors (90%) by standard diagnostic testing, including karyotype and/or FISH for *DDIT3 locus*. The CNAs detected most often in MLPS were losses of 6q (8/20; 40%) and gains of 1q (6/20; 30%) (Fig. 3c). The minimal overlapping region of 36 Mbp at 6q14.1q22.31 covered a total of 175 genes, including four genes from the *Cancer Gene Census* database (*PRDM1, FOX03, ROS1, GOPC*), while gains of 1q25.1q32.2 of 34 Mbp encompassed a total of 269 genes, among which six genes are known to be involved in cancer pathogenesis (*ABL2, TPR, CDC73, MDM4, ELK4, SLC45A3*). Among other CNAs present in MLPS, trisomy of chromosome 8 (3/20; 15%), 13 (2/20; 10%) and a loss of chromosome 16q (2/20; 10%) were present in more than one case each. No statistically significant correlations with the type and the location of tumors were found, but additional copies of chromosome 1 were more frequently observed in tumors with increased genomic complexity (*p* = 0.035; Mann-Whitney test).

## Discussion

In the present study, we have performed the comparison of the genomic profiles established by means of simultaneous classical and molecular cytogenetics analyses in a large series of three LPS subtypes (WDLPS, DDLPS and MLPS). In addition, we estimated the efficacy of implementation the array-CGH analyses into the panel of routine diagnostic procedures in LPS. On the other hand, evaluation of the prognostic significance of particular chromosomal abnormalities on clinical outcome of the patients is beyond the scope of current research.

The significantly higher resolution of array-CGH over conventional karyotyping allowed to detect CNAs in tumors with greater sensitivity and precision. In the current study, six tumors with apparently normal genomic profiles established by conventional technique were found to harbor unbalanced chromosomal aberrations. Certain marker and/or ring chromosomes were large enough to be identified through light microscope, however in most cases application of the molecular method allowed for identification of a number of additional events. Array-CGH screening test facilitated identification of the possible origin of the marker/ring chromosomes in 67% (31/46) of WDLPS and DDLPS cases.

Not only small genomic imbalances were identified, but this technique also allowed for estimation of the amplification frequency of specific genes (Fig. 2). Amplification of 12q, commonly observed in WDLPS and DDLPS tumors, covers *loci* of several oncogenes, including *MDM2, CDK4* and *HMGA2* that are proposed to play the role in the molecular pathogenesis of both subtypes [16]. In the current study, *MDM2, CDK4* and *HMGA2* amplifications were found in ~92% of samples what is in line with previously reported incidence [1, 4, 9, 10, 17]. Moreover, recently published data have demonstrated that the 12q14.1q21.1 amplicon may contain the other genes, presumably involved in LPS pathogenesis, such as *FRS2* (12q15) or *CPM* (12q15) [11, 17–19]. In our series, the frequency of high – level amplifications of the *FRS2* and *CPM* genes was somehow lower than previously reported (88% and 85% vs. 97% and 89%, respectively).

According to the guidelines of the *European Sarcoma Network Working Group* the genetic testing should be the mandatory part of the pathological diagnosis of soft tissue sarcomas (2014) [20]. As pointed out by Italiano et al. (2016), molecular genetic screening facilitated establishing accurate diagnosis in 14% (53/384) sarcoma cases. The highest rate of misdiagnoses prior to molecular testing was observed in the DDLPS cohort (23%, 7/30) [21]. In the current study, initially four WDLPS tumors (6%) were wrongly classified, but in light of the array-CGH profiling they were eventually diagnosed as DDLPS (Fig. 1b  and Additional file 2: Table S2). The distinction between WDLPS and DDLPS is challenging, because both are characterized by the presence of ring and marker chromosomes and 12q14q15 amplification, established routinely by conventional karyotyping and FISH, respectively (Fig. 3). The application of array-CGH allowed to identify the specific set of genomic imbalances in DDLPS (Table 1; Fig. 3b) that could be used as specific marker in differential diagnosis with WDLPS. Losses of chromosomes 11 and 13, associated with increased genomic complexity of tumors, were observed exclusively in DDLPS. These CNAs encompass a number of cancer-associated genes, among which a few have already been proposed as candidate genes in the pathogenesis of soft tissue sarcomas, i.e. *RB1* [22]. It has been demonstrated that 16% of lipomas, benign fatty tumors, have harbored 13q14 losses, while in spindle cell lipomas the frequency of this aberration is almost 100% [23, 24]. Moreover, the coexistence of retinoblastoma and lipoma/liposarcoma was observed in sporadic cases, even though the role of the *RB1* gene in their pathogenesis and differentiation process remains still unknown [25, 26]. The other CNAs that are nearly specific to DDLPS included gains of chromosome 14q and losses of chromosome 6.

Overall, genomic imbalances were far more numerous in the DDLPS tumors than in WDLPS (5x) and MLPS (2.5x). Previously, Crago et al. (2012) have shown that WDLPS and DDLPS had more CNAs, affecting 5.7% and 21% of the genome, respectively [10]. The difference in the reported genomic complexity between theirs and the current study may be explained by the localization of tumors. Most of the neoplasms (89%) presented in their study were located in the retroperitoneum that is associated with poorer prognosis, compared with only 39% of such tumor location in our study.

So far, trisomy of chromosome 8 [22, 27–29] and 13q gains [30] have been reported as the CNAs with the highest prevalence in MLPS. The additional copies of chromosome 13 have been suggested to correlate with poorer prognosis of MLPS patients [30]. However, these observations were not in line with our results; gains of chromosome 8 and 13 were identified only in three and two tumors, respectively. The most frequently involved chromosomal regions in MLPS were losses of chromosome 6 and gains of chromosome 1 (Fig. 3c).

Most neoplasm disorders are characterized by chromosomal instability (CIN) that is defined as a genomic instability with observed high rate of chromosomal losses and/or gains. Even though CIN is typical for the vast majority of human cancers, its exact contribution to tumor progression is still deliberated [31]. An increasing number and size of genomic alterations from a primary tumor to its metastasis was confirmed by our studies. Nearly two-fold increase in genomic complexity in recurrences and metastases was observed, compared with the primary tumors. Moreover, array-CGH analysis revealed that losses of chromosome 6 and gains of chromosome 5 were observed more frequently in the recurrences and metastases than in the primary LPS tumors regardless of their histological subtype.

The number of technical difficulties faced during cytogenetic chromosome preparations, such as high incidence of cell culture failures, contamination or normal cell growth may be overcome by using array-CGH. Array-CGH appears as a less time-consuming (analysis can be performed within 72 h) and a cost-effective genome-wide screening tool. Notwithstanding the aforesaid, array-CGH has several limitations. First of all, it does not detect balanced translocations, inversions or point mutations. In order to assess the presence of the specific balanced translocations t(12;16) or t(12;22) in MLPS tumors, classical karyotyping and/or targeted FISH has to be nonetheless performed. Furthermore, fresh frozen material is the preferred source of DNA for array-CGH analyses, because paraffin- embedded tissue (FFPE) specimens often increase experimental noise [32] leading to an elevated rate of false positive CNAs calls. That is contrary to the standard pathological procedures that prefer FFPE over fresh-frozen samples. Moreover, tumor DNA may be contaminated with DNA from nonneoplastic cells, and even the tumor cells are histologically and genetically heterogeneous. To avoid masking of acquired aberrations by normal tissue DNA it has been strongly suggested to ensure at least 25% of tumor cells in sample [33]. Constitutional normal DNA from the patient with tumor sample has been recommended as reference in array-CGH analysis, however in clinical practice it is difficult to achieve. Accordingly, to distinguish clonal from constitutional aberrations each abnormality should be verified in the databases of polymorphic, benign copy number variations (CNVs). In this study, the homozygous deletion at 8p11.23p11.22, encompassed *ADAM3a* and *ADAM5* genes, was observed in 42% cases of WDLPS tumors. Losses of 8p11.23p11.22 have been also identified in 16% of pediatric high-grade gliomas [34] and 7% of the non-small cell lung cancer samples that have been suggested to be correlated with poorer prognosis of these patients [35]. However, we demonstrated that this aberration was observed in both normal tissue and the tumor sample, implying the possibility of occurrence of nonpathogenic copy number variation (CNV) what is consistent with the findings of Li et al. [36]. Hence, it is essential to accurately identify somatic aberrations in cancer profile genome to exclude the critical errors that may cause data to be misleadingly interpreted.

## Conclusion

In conclusion, the application of array-CGH allowed to delineate clearly the genomic profiles of WDLPS, DDLPS and MLPS that reflect biological differences between these tumors. We demonstrate that knowledge of the genome profile along with the detailed histological examination may help to reduce misdiagnoses of LPS subtypes. Specific set of genomic changes, established by array-CGH in DDLPS may facilitate diagnostic dilemma. In order to assess the significance of these alterations in LPS patients, further extensive studies on well-defined larger cohorts and correlations with clinical data should be conducted. In addition, we provide the evidence that array-CGH is an appropriate complementary method to standard diagnostic approach of conventional karyotyping and FISH, however the implementation of high-resolution profiling in routine diagnostic practice should be undertaken selectively.

## Additional files

Additional file 1: Table S1. Clinicopathological data of the liposarcoma samples included in the study. (DOCX 21 kb)
Additional file 2: Table S2. Karyotypes and DNA copy number changes established by array-CGH in 66 liposarcoma tumors. (DOCX 44 kb)

## References

[1] Coindre JM, Pedeutour F, Aurias A (2010). Well-differentiated and dedifferentiated liposarcomas. Virchows Arch.

[2] Miettinen M, Miettinen M (2010). Atypical lipomatous tumor and liposarcomas. Modern soft tissue pathology.

[3] Bridge JA (2014). The role of cytogenetics and molecular diagnostics in the diagnosis of soft-tissue tumors. Mod Pathol.

[4] Conyers R, Young S, Thomas DM. Liposarcoma: molecular diagnostics and therapeutics. Sarcoma. 2011;483154. 10.1155/2011/483154 PMC3021868 21253554

[5] Dei Tos AP (2014). Liposarcomas: diagnostic pitfalls and new insights. Histopathology.

[6] Mastrangelo G, Coindre JM, Ducimetiere F, Dei Tos AP, Fadda E, Blay JY, Buja A, Fedeli U, Cegelon L, Frasson A, Ranchere-Vince D, Montesco C, Ray-Coquard I, Rossi CR (2012). Incidence of soft-tissue sarcoma and beyond: a population-based prospective study in 3 European regions. Cancer.

[7] Limon J, Turc-Carel C, Dal Cin P, Sandberg AA (1986). Recurrent chromosome translocations in liposarcoma. Cancer Genet Cytogenet.

[8] Bode-Lesniewska B, Brigerio S, Exner U, Abdou MT, Moch Z, Zimmermann DR (2007). Relevance of translocation type in myxoid liposarcoma and identification of a novel EWSR1-DDIT3 fusion. Genes Chromosomes Cancer.

[9] Tap WD, Eilber FC, Ginther C, Dry SM, Reese N, Barzan-Smith K, Chen HW, Wu H, Eilber FR, Slamon DJ, Anderson L (2011). Evaluation of well-differentiated/de-differentiated liposarcomas by high-resolution oligonulceotide array-based comparative genomic hybrydization. Genes Chromosomes Cancer.

[10] Crago AM, Socci ND, DeCarolis P, O’Connor R, Taylor BS, Qin LX, Antonescu CR, Singer S (2012). Copy numer losses define subgroups of dedifferentiated liposarcomas with poor prognosis and genomic instability. Clin Cancer Res.

[11] Kanojia D, Nagata Y, Garg M, Lee DH, Sato A, Yoshida K, Sato Y, Sanada M, Mayakonda A, Bartenhagen C, Klein HU, Doan NB, Said JW, Mohith S, Gunasekar S, Shiraishi Y, Chiba K, Tanaka H, Miyano S, Myklebost O, Yang H, Dugas M, Meza-Zepeda LA, Silberman AW, Forscher C, Tyner JW, Ogawa S, Koeffler HP (2015). Genomic landscape of liposarcoma. Oncotarget.

[12] Miller SA, Dykes DD, Polesky HF (1988). A simple salting out procedure for extracting DNA from human nucleated cells. Nucleic Acids Res.

[13] Ronowicz A, Brzeskwiniewicz M, Madanecki P, Buckley PG, Orlowska E, Ochocka R, Limon J, Piotrowski A (2012). Regeneration of comparative genomic hybridization oligonucleotide microarrays with dimethylurea. Anal Biochem.

[14] Database of Genomic Variants. http://dgv.tcag.ca/dgv/app/home. Accessed May 2015.

[15] Limon J, Dal Cin P, Sandberg AA (1986). Application of long-term collagenase dissaggregation for the cytogenetic analysis of human solid tumors. Cancer Genet Cytogenet.

[16] Pedeutour F, Forus A, Coindre JM, Berner JM, Nicolo G, Michniels JF, Terrier P, Ranchere-Vince D, Collin F, Myklebost O, Turc-Carcel C (1999). Structure of the supernumerary ring and giant rod chromosomes in adipose tissue tumors. Genes Chromosomes Cancer.

[17] Wang X, Asmann YW, Erickson-Johnson MR, Oliveira JL, Zhang H, Moura RD, Lazar AJ, Lev D, Bill K, Lloyd RV, Yaszemski MJ, Maran A, Oliveira AM (2011). High-resolution genomic mapping reveals consistent amplification of the fibroblast growth factor receptor substrate 2 gene in well-differentiated and dedifferentiated liposarcoma. Genes Chromosomes Cancer.

[18] Erickson-Johnson MR, Seys AR, Roth CW, King AA, Hulshizer RL, Wang X, Asmann YW, Lloyd RV, Jacob EK, Oliveira AM (2009). Carboxypeptidase M: a biomarker for the discrimination of well-differentiated liposarcoma from lipoma. Mod Pathol.

[19] Zhang K, Chu K, Wu X, Gao H, Wang J, Yuan YC, Loera S, Ho K, Wang Y, Chow W, Un F, Chu P, Yen Y (2013). Amplification of FRS2 and activation of FGFR/FRS2 signaling pathway in high-grade liposarcoma. Cancer Res.

[20] ESMO/European Sarcoma Network Working Group (2014). Soft tissue and visceral sarcomas: ESMO Clinical Practice Guidelines for diagnosis, treatment and follow-up. Ann Oncol.

[21] Italiano A, Di Mauro I, Rapp J, Pierron G, Auger N, Alberti L, Chibon F, Escande F, Voegeli AC, Ghnassia JP, Keslair F, Lae M, Ranchere-Vince D, Terrier P, Baffert S, Coindre JM, Pedeutour F (2016). Clinical effect of molecular methods in sarcoma diagnosis (GENSARC): a prospective, multicentre, observational study. Lancet Oncol.

[22] Mandahl N and Mertens F. Soft tissue tumors. In: Heim S and Mitelman F, editors. Cancer cytogenetics: Chromosomal and molecular genetic aberrations of tumor cells. Chichester: John Wiley & Sons, Ltd; 2015. p. 583–589.

[23] Dahlen A, Debiec-Rychter M, Pedeutour F, Domanski HA, Hoglund M, Bauer HC, Rydholm A, Sciot R, Mandahl N, Mertens F (2003). Clustering of deletions on chromosome 13 in benign and low-malignant lipomatous tumors. Int J Cancer.

[24] Bartuma H, Nord KH, Macchia G, Isaksson M, Nilsson J, Domanski HA, Mandahl N, Mertens F (2011). Gene expression and single nucleotide polymorphism array analyses of spindle cell lipomas and conventional lipomas with 13q14 deletion. Genes Chromosomes Cancer.

[25] Li FP, Abramson DH, Tarone RE, Kleinerman RA, Fraumeni JF, Boice JD (1997). Hereditary retinoblastoma, lipoma and second primary cases. J Natl Cancer Inst.

[26] Genuardi M, Klutz M, Devriendt K, Caruso D, Stirpe M, Lohmann DR (2001). Multiple lipomas linked to an RB1 gene mutation in a large pedigree with low penetrance retinoblastoma. Eur J Hum Genet.

[27] Szymanska J, Tarkkanen M, Wiklund T, Blomgvist C, Asko-Seljavaara S, Tukiainen E, Elomaa I, Knuutila S (1996). Gains and losses of DNA sequences in liposarcomas evaluated by comparative genomic hybridization. Genes Chromosomes Cancer.

[28] Parente F, Grosgeorge J, Coindre JM, Terrier P, Vilain O, Turc-Carel C (1999). Comparative genomic hybridization reveals novel chromosome deletions in 90 primary soft tissue tumors. Cancer Genet Cytogenet.

[29] Ohguri T, Hisaoka M, Kawauchi S, Sasaki K, Aoki T, Kanemitsu S, Matsuyam A, Korogi Y, Hashimoto H (2006). Cytogenetic analysis of myxoid liposarcoma and myxofibrosarcoma by array-based comparative genomic hybridization. J Clin Pathol..

[30] Schmidt H, Bartel F, Kappler M, Wurl P, Lange H, Bache M, Holzhausen HJ, Tauber H (2005). Gains of 13q are correlated with a poor prognosis in liposarcoma. Mod Pathol.

[31] Heng HH, Bremer SW, Stevens JB, Horne SD, Liu G, Abdallah BY, Ye KJ, Ye CJ (2013). Chromosomal instability (CIN): what it is and why it is crucial to cancer evolution. Cancer Metastasis Rev.

[32] Nakao K, Oikawa M, Arai J, Mussazhanova Z, Kondo H, Schichijo K, Nakashima M, Hayashi Y, Yoshiura K, Hatachi T, Nagayasu T (2013). A predictive factor of the quality of microarray comparative genomic hybridization analysis for formalin-fixed paraffin-embedded archival tissue. Diagn Mol Pathol.

[33] Cooley LD, Lebo M, Li MM, Slovak ML, Wolff DJ (2013). American College of Medical Genetics and Genomics technical standards and guidelines: microarray analysis for chromosome abnormalities in neoplastic disorders. Genet Med.

[34] Barrow J, Adamowicz-Brice M, Cartmill M, MacArthur D, Lowe J, Robson K, Brundler MA, Walker DA, Coyle B, Grundy R (2011). Homozygous loss of ADAM3A revealed by genome - wide analysis of pediatric high - grade glioma and diffuse intrinsic pontine gliomas. Neuro Oncol.

[35] Wang Y, Zhang Y, Wu L (2011). Homozygous deletion of ADAM3A revealed by genome - wide analysis early - stage NSCLS in China showed to be correlated with poor prognosis. J Clin Oncol.

[36] Li A, Liu Y, Zhao Q, Feng H, Harris L, Wang M (2014). Genome-wide identification of somatic aberrations from paired normal-tumor samples. Plos One.

