# Supplementary material for: Application of high-resolution genomic profiling in the differential diagnosis of liposarcoma
Source: Mol Cytogenet. 2017 Mar 16;10:7. doi: 10.1186/s13039-017-0309-5 (PMC5356274; doi:10.1186/s13039-017-0309-5)
Supplement: Additional file 1: Table S1. — Clinicopathological data of the liposarcoma samples included in the study. (DOCX 21 kb) [file 13039_2017_309_MOESM1_ESM.docx]

**Table S1**. Clinicopathological data of the liposarcoma samples included in the study.

|  | | **WDLPS**  **(n=23 tumors from 18 patients)** | **DDLPS**  **(n=23 tumors from 16 patients)** | **MLPS**  **(n=23 tumors from 19 patients)** | **Total**  **(n=69 tumors from 53 patients)** |
| --- | --- | --- | --- | --- | --- |
| **Patients number** | Age, median (range)  Gender, n (%)  Female  Male | 50 (32-65)  7 (39%)  11 (61%) | 50 (37-78)  9 (56%)  7 (44%) | 51 (37-82)  8 (42%)  11 (58%) | 50 (32-82)  24 (45%)  29 (55%) |
| **Tumors number** | Origin, n (%)  Primary (P)  Reccurence (R)  Metastasis (M)  Location, n (%)  Retroperitoneum  Extremities  Other | 14 (61%)  9 (39%)  0  12 (52%)  9 (39%)  2 (9%) | 5 (22%)  15 (65%)  3 (13%)  9 (39%)  2 (9%)  12 (52%) | 9 (39%)  7 (30%)  7 (30%)  6 (26%)  12 (52%)  5 (22%) | 28 (41%)  31 (45%)  10 (14%)  27 (39%)  23 (33%)  19 (28%) |
