# Supplementary material for: Application of high-resolution genomic profiling in the differential diagnosis of liposarcoma
Source: Mol Cytogenet. 2017 Mar 16;10:7. doi: 10.1186/s13039-017-0309-5 (PMC5356274; doi:10.1186/s13039-017-0309-5)
Supplement: Additional file 2: Table S2. — Karyotypes and DNA copy number changes established by array-CGH in 66 liposarcoma tumors. (DOCX 44 kb) [file 13039_2017_309_MOESM2_ESM.docx]

**Table S2**. Karyotypes and DNA copy number changes established by array-CGH in 66 liposarcoma tumors.

| **Case no.** | **Origin** | **Karyotype** | **Losses** | **Gains** | | **% genome altered** |
| --- | --- | --- | --- | --- | --- | --- |
|  |  |  |  | **Chromosome 12** | **Other chromosomes** |  |
| 1 | P | 47,XX,+r[8]/46,XX[5] | - | *12q13.12, 12q13.3q14.1, 12q14.1, 12q14.2, 12q14.3, 12q15, 12q15q21.1, 12q21.1,* 12q21.2q21.31, 12q21.32, 12q22, 12q23.1 | *1q21.1*, 15q26.2q26.3 | 0.69 |
| 2 | P | 48,XY,+r1,+r2[5] | 10q23.31 | *12q14.1, 12q14.3q15*, 12q15q21.1, 12q21.2q21.31, *12q21.31, 12q21.33*, 12q22, *12q23.1, 12q23.3, 12q24.31* | 2q32.2q32.3, 2q34, *3p14.1, 3p13, 3p12.3, 3p11.2p12.2, 3p12.1*, 3p12.1p11.2, 3q27.3q28, *5p13.1, 6q23.3, 6q24.1, 6q24.2, 6q24.3q25.1, 6q25.1, 6q25.2, 6q25.3,* 13q32.1q32.2, 13q33.2, 13q33.2q33.3 | 1.27 |
| 3 | P | 46,XY,-6.+r[7].ish r(wcp12+)[4]/  81~84,idemx2,inc[cp4]/46,XY[3] | - | *12q13.3q14.1, 12q14.1, 12q14.2, 12q14.3, 12q14.3q15, 12q15, 12q21.1, 12q21.1q21.2, 12q21.2, 12q21.31, 12q21.33, 12q22q23.1, 12q23.1, 12q23.2* | *11q22.1* | 0.60 |
| 4 | P | 45~47,mar,inc[cp8] | - | 12p13.32, 12p13.31, 12p11.22p11.1, *12q12, 12q13.13, 12q14.1, 12q14.2, 12q14.3, 12q15q21.1, 12q21.1, 12q21.2, 12q21.31, 12q21.32, 12q21.33, 12q22, 12q23.1* | 1p36.33p36.32 1p36.22p36.21 1p36.13p36.12, 2p12p11.2, *3p25.3, 3p24.3, 3p22.2, 3p14.1, 3p12.3, 3p11.2p11.1, 3q12.2,*7p22.3p22.1, 7p15.1, 7p14.3, 7p12.1, 9q33.3q24.11, 19p13.3p13.2, 19p13.2, 19p13.2p13.13, 19p13.13p13.12, 19q13.11, 19q13.11q13.12, 19q13.12, 19q13.13, 19q13.2, 19q13.31, 19q13.32, 19q13.41q42, 20p13, 20q13.31, 20q13.32 | 1.80 |
| 5 | P | 48,XY,+r1,+r2[7] | - | *12q12, 12q13.3q14.1, 12q14.2, 12q14.3q21.31* | - | 0.40 |
| 6 | P | 42~52,mar1,mar2,inc[cp5] | 9p24.3p13.3 | *12q13.3q14.1, 12q14.1q14.2, 12q14.3q15, 12q15q21.1, 12q21.1q21.2, 12q21.31q21.32, 12q21.32q21.33* | 1q21.1, 1q21.2, 1q21.2q21.3, 1q21.3q22, 1q23.2q23.3, 1q23.3, 1q24.1q25.1, 1q25.2, 7q36.3, 19p13.3p12 | 1.86 |
| 7 | P | 46~48,XX,+r1,+r2,+mar,inc[cp8] | - | 12q12, *12q13.11, 12q13.12*, 12q13.13, *12q13.3, 12q14.1, 12q14.2, 12q14.3, 12q15, 12q21.1, 12q21.2, 12q21.31, 12q21.32, 12q21.33, 12q22, 12q23.1,* 12q23.2, 12q23.3, *12q24.21, 12q24.23, 12q24.31, 12q24.32* | 2q12.1, 3q27.1q28, 18q11.1, 18q11.2q12.1, 18q12.1, 18q12.2q21.2, 18q21.2q21.32, 18q21.32, 19p13.3p12, 19q13.12 | 3.42 |
| 8 | P | 46,XY | 1q25.3q31.3, 1q31.1q32.1, 1q32.1, 1q32.2, 1q32.2q32.3, 1q41q44, 4q26, 4q32.1q32.2, 4q34.3q35.2, 5q11.1q31.2, 15q13.2, 15q13.3 15q14q21.1, 22q12.1q13.33 | 12q12, *12q14.1, 12q14.3, 12q15, 12q21.1q21.33, 12q22q23.1, 12q23.1, 12q23.1q23.2, 12q23.3, 12q24.11,* 12q24.13, *12q24.21, 12q24.32q24.33* | 1q12q24.1, *1q24.1q25.1*, 1q25.11q31.3, 1q32.1, 1q32.1q32.2, *1q32.2,* 1q32.3, 1q32.3q41, *4q23, 4q24*, 5p15.33p13.1, 22q11.21, 22q11.21q12.1 | 10.29 |
| 9 | P | 46,XY | - | - | - | 0 |
| 10 | P | 46,XX | - | 12p13.33, *12p12.3, 12p12.1, 12p11.21,* 12q12, 12q13.13, 12q13.2, *12q14.1, 12q14.2q14.3, 12q15, 12q15q21.1, 12q21.1q21.2, 12q21.2, 12q21.31, 12q21.32, 12q21.33, 12q22*, 12q23.1, 12q23.2, 12q23.3, *12q24.31* | 1q21.2q21.3 , 1q21.3, *1q23.2, 1q23.3*, 1q23.3q24.2, 1q24.2, 1q24.2q24.3, 1q24.3, 1q25.2, 1q31.3, 5p15.33 | 1.09 |
| 11 | P | 42~50,XY,+r1,+r2,inc[cp3]/  92~97,idemx2,+mar,inc[cp3] | - | *12q13.13, 12q13.2, 12q13.3, 12q14.1, 12q14.2, 12q14.3, 12q15, 12q21.1, 12q21.2, 12q21.31, 12q23.3* | *5p15.33, 5p15.33p15.32, 5p15.32, 5p15.2, 5p15.1p14.3, 5p14.3, 5p14.2 5p14.2p14.1, 5p14.1, 5p14.1p13.3, 5p13.3, 5p13.2, 5p13.1, 5p13.1p12, 5p12, 14q13.1q21.1, 14q21.2q21.3, 14q22.1* | 1.08 |
| 12 | R | 25~98,X,t(2;3;?)(p26;q?28;?),add(18)(q13),  add(19)(q13)[2],r1[3],r2[4]inc[cp12] | - | *12q13.2, 12q13.3q14.2, 12q14.3q21.1, 12q21.1q21.2, 12q21.31* | *1p22.3, 1p12, 1q12q21.1, 1q21.2, 1q21.2q21.3, 1q21.3q23.1, 1q23.1, 1q23.2, 1q23.2q23.3, 1q23.3, 1q23.3q24.1, 1q24.3, 1q32.1, 1q43 2p15.2,*2p12, 2q21.2, 2q21.2q21.3, 2q21.3, 2q22.1, 2q24.1, 2q33.3, *5q33.3, 11q21, 11q22.1, 11q22.1q22.2, 11q22.2, 14q23.3, 14q32.13q32.2, 14q32.31q32.32, 14q32.32, 14q32.33, 15q14, 15q15.1, 15q15.2q15.3, 15q15.3q21.1, 15q21.1, 16p13.3, 16p13.13, 16q22.1q22.2* | 1.60 |
| 13 | R | 46~48,XX,+der(19)t(5;19)(q13;q13),  +r1,+r2,inc[cp10]/90-92,idemx2,inc[cp4] | - | *12q12, 12q13.13, 12q13.3q14.1, 12q14.1, 12q14.2, 12q14.3, 12q15, 12q21.1, 12q21.2, 12q21.2q21.31, 12q21.31, 12q21.32, 12q21.33, 12q22, 12q23.1, 12q23.2, 12q24.13* | 5p15.33 5p15.33p15.32, 5p15.31p15.2, 5p15.2p14.3, 5p14.3p14.2, 5p14.2, 5p14.2p14.1, 5q14.2, 5q14.2q14.2, 5q15, 5q21.1, 5q23.2, *22q11.21, 22q12.2q12.3, 22q12.3* | 1.46 |
| 14 | R | 45~50,XX,+r,+mar1,+mar2,+mar3,inc[cp7] | - | *12q12, 12q13.12, 12q13.13, 12q14.1, 12q14.2, 12q14.3, 12q15, 12q21.1, 12q21.2, 12q21.31, 12q21.32, 12q21.33* | 6p25.3p11.1, *6q11.1, 6q12q13, 6q13, 6q25.3q26, 6q27, 14q12, 14q13.1, 14q13.2, 14q13.3, 14q21.2, 14q21.3, 14q21.3q22.1, 14q22.1, 14q22.2, 14q22.3, 14q23.1, 14q23.2q23.3, 14q24.1, 14q24.3, 14q32.12, 14q32.2, 14q32.31, 16q12.1, 16q21, 16q22.1, 16q22.3, 16q23.1* | 4.32 |
| 15 - 1 | R | 47,XY,+r[3]/75-90,idemx2,inc[cp3] | - | *12q13.11, 12q13.13, 12q13.2, 12q14.1, 12q14.2, 12q14.2q14.3, 12q14.3, 12q14.3q15, 12q15, 12q15q21.1, 12q21.1*, 12q21.2 | *5p15.2, 5p15.1, 5p14.3, 5p14.2, 5p14.1, 5p13.3, 5p13.2,* 6q25.1q25.2 | 0.76 |
| 15 - 2 | R | 47,XY,+r[8]/88-96,idemx2[cp4]/46,XY[7] | - | *12q13.11, 12q13.13, 12q13.2, 12q14.1, 12q14.2, 12q14.2q14.3, 12q14.3, 12q14.3q15, 12q15, 12q15q21.1, 12q21.1*, 12q21.2, 12q21.31, 12q21.32, 12q24.32 | *5p15.2, 5p15.1, 5p14.3, 5p14.2, 5p14.1, 5p13.3, 5p13.2,*6q25.1q25.2 | 1.00 |
| 15 - 3 | R | 44~45,r1,r2.ish r1(wcp12+),  r2(wcp12+),inc[cp5]/  92~94,idemx2,+r3,+mar, inc[cp8] | - | *12q13.11, 12q13.13, 12q13.2, 12q14.1, 12q14.2, 12q14.2q14.3, 12q14.3, 12q14.3q15, 12q15, 12q15q21.1, 12q21.1,* 12q21.2 | *5p15.2, 5p15.1, 5p14.3, 5p14.2, 5p14.1, 5p13.3, 5p13.2, 5q14.2, 5q14.3* | 0.52 |
| 15 - 4 | R | 42~48,XY,+r1,+r2,inc[cp6]/  71~82,r1,r2,inc[cp3]/46,XY[5] | - | *12q13.11, 12q13.13, 12q13.2, 12q14.1, 12q14.2, 12q14.2q14.3, 12q14.3, 12q14.3q15, 12q15, 12q15q21.1, 12q21.1*, 12q21.31, 12q24.32 | *4p15.33, 5p15.2, 5p15.1, 5p14.3, 5p14.2, 5p14.1, 5p13.3, 5p13.2 6q14.2, 6q16.2q16.3, 6q21, 6q22.31q22.32, 6q22.33, 6q23.1, 6q23.2q23.3, 6q23.3q24.2, 6q24.3q25.3, 6q26,*19p13.3p13.1, 19q12 | 1.93 |
| 15 - 5 | R | 31~46,r,mar,inc[cp10] | - | *12q13.11, 12q13.13, 12q13.2, 12q14.1, 12q14.2, 12q14.2q14.3, 12q14.3, 12q14.3q15, 12q15, 12q15q21.1, 12q21.1*, 12q21.2 | 4p16.3p12, *5p15.2, 5p15.1, 5p14.3, 5p14.2, 5p14.1, 5p13.3, 5p13.2*, 19p13.3p13.1 | 2.75 |
| 16 - 1 | P | 38~48,r1,r2,mar[3],2~5dmin,inc[cp10] | - | 12p13.33p13.31, *12q13.3q14.1, 12q14.1, 12q14.2, 12q14.3, 12q15, 12q21.1, 12q21.2, 12q21.33, 12q21.33q22, 12q22* | *3p14.1, 3p12.3, 17p13.1, 17p12p11.2* | 0.76 |
| 16 - 2 | P | 44~47,r1,r2,mar[3],2~3dmin,inc[cp8] | - | 12p13.33p13.32, 12p3.32, *12q13.3q14.1, 12q14.1, 12q14.2, 12q14.3, 12q15, 12q21.1, 12q21.2, 12q21.33, 12q21.33q22, 12q22* | *3p14.1, 3p12.3,* 8p23.3p23.2, 8p11.21, 8q11.1q12.3, 8q12.3q13.3, 8q13.3, 8q21.11, 8q21.11q21.12, 8q21.12, 8q21.12q21.13, 8q21.13, 8q21.3, 8q22.1*,* 13q13.3q14.11, 13q21.33, 13q33.1q33.2, 13q33.3q34*, 17p12, 17p12p11*, 19q12, 19q13.12q13.2, 19q13.32, 19q13.33q13.43 | 2.63 |
| 17 | P | 47~50,XY,+r1,+r2,+r3,inc[cp7] | 19q12q13.2 | *12q13.2, 12q13.3, 12q14.1, 12q14.2, 12q14.3, 12q15q21.31, 12q21.32, 12q21.33, 12q24.21* | *9q34.3, 11q24.2, 13q14.11q14.12, 18p11.31* | 0.96 |
| 18 | P | 42~52,mar1,mar2,inc[cp5] | 19p13.3p13.11 | *12p13.32, 12q13.2, 12q14.1, 12q14.2, 12q14.3q15, 12q21.1, 12q21.2, 12q21.31, 12q21.33, 12q23.1* | 4p16.1p12, 6q23.3 6q24.1q25.2 , *13q33.3q34* | 3.00 |
| 19* | P | 90~120,r1x2,r2,inc[cp4]/46,XY[6] | 11q22.3q25, 13q14.12q34, 17q11.2q12 | - | 2q32.2q37.3, 4q34.1q35.2, 11p15.5p15.4, 11p15.3p12, 11q12.2q14.1, 11q22.1q22.3, 13q12.12q14.12, 17p12p11.2, 17q11.1q11.2, 17q12q21.31, 17q21.32q21.33, 19p13.3p13.11 | 9.18 |
| 20 | P | 63~71,mar1,mar2,mar3,mar4,inc[cp5] | 5p15.33p11, 9p24.3p11.1, 9q12q13, 11q11q25, 12p13.33p11.22, 22q13.31q13.33 | *12q12, 12q14.1, 12q14.2, 12q14.3, 12q14.3q15, 12q15q21.1, 12q21.1, 12q21.2, 12q21.31, 12q21.33, 12q22, 12q23.1, 12q24.22q24.31* | 4p16.3p15.1, 8p23.3p23.2, 8q13.2q23.3, 8q24.13, 8q24.21, 8q24.3, 9q21.13q22.2, 9q22.33q31.3 | 1.26 |
| 21 | P | 46,XY | 1q25.1q43, 3p25.1p24.3, 4q32.2, 4q32.3, 4q33q34.1, 4q34.3q35.2, 8p23.2p11.21, 13 | *12q14.1, 12q15* | 5, 7p22.1p11.1, 7q11.1q36.3, 14, 19, 20 | 24.12 |
| 22 | R | 32~46,XX, add(6)(p25),del(6)(q13),  add(11)(q21),der(14;18)(q10;q10),  der(17)t(6;17)(q13;q25),+mar1,+mar2,inc [cp11] | 6p23p22.2, 14q21.1, 14q21.2q21.3, 14q21.3q22.3, 14q22.3, 14q23.1, 15q12q14, 15q26.1q26.3 | *12q12, 12q13.2q13.3, 12q14.1, 12q14.3, 12q15q21.1, 12q21.1, 12q21.2, 12q21.31, 12q21.32q22, 12q22, 12q23.1* | 5q14.3, 5q22.2q23.1, 5q23.2, 7p22.3p21.3, 7p21.3p21.2, 14q11.2, 14q21.1q21.2, 14q21.3, 14q22.3, 14q22.3q23.1, 14q23.1, 14q32.13q32.2, 14q32.33, 19q13.11, 19q13.13, 19q13.2 | 3.03 |
| 23 | R | 38~45,r1,mar1,mar2,inc[cp5]/  71~84,r1,r2,mar1,inc[cp4] | 21q21.2q21.3 | *12q13.2, 12q13.3q14.1, 12q14.1q14.2, 12q14.3q21.1, 12q21.1q21.2, 12q21.31* | 1q21.1, 1q21.2q21.3, 1q21.3, 1q23.1, 1q23.2, 1q23.2q24.1, 1q24.2q24.3, 1q24.3, 1q42.12, 1q43, 1q43q44, 2p15p14, 2p12, 2q21.2q21.3, 2q21.3, 2q22.1, 2q24.1, 2q33.3, 5q33.3, 11q21, 11q22.1q22.2, 14q23.3, 14q32.2, 14q32.31q32.32, 14q32.33, 15q14, 15q15.1, 15q15.2q15.3, 15q15.3, 15q15.3q21.1, 16p13.3, 16p13.13 | 1.43 |
| 24 | R | 68~74,XY,del(1)(q11),dic(1;9)t(p22;p13),  der(2)t(2;4)(p13;q12),del(3)(p21)x2,  del(4)(p11),der(6)t(2;6)(p11;p11),del(7)(p15),del(7)(q32),der(7)t(7;8)(p11;p11),del(8)(p11),  del(9)(p13),del(9)(p13),add(9)(q34),  add(11)(p13),add(11)(p15),add(11)(q13),  del(12)(p11),der(16)t(9;16)(q11;p11),  der(16)t(15;16)(q11;q11)x2,  der(17)t(3;17)(q21;p13),i(18)(q10),  add(19)(q13),der(19)t(17;19)(q21;q13),  der(?)t(10;?)(q11;?),inc[cp7] | 2q21.1q37.3, 4p16.2p14, 6p25.3p12.3, 8q23.1q24.3, 9p24.3p21.3, 9p21.2p13.3, 10q11.22q23.33 , 11p15.5p12, 11q23.2q24.2, 13, 18p11.31p11.21, 18q22.1q23, 20p13p12.1, 21q21.2q21.2 | *12p11.22p11.1, 12q12q13.13, 12q14.1, 12q14.3, 12q14.3q15, 12q15q21.1, 12q21.1, 12q21.2, 12q21.31, 12q21.32, 12q21.33q22, 12q23.1* | 1q21.1q25.3, 1q31.2q32.1, 1q32.3q41, 5p15.2p15.1, 5p12p11, 5q11.1q12.2, 8q11.21q22.3, 9p21.3p21.2, 9p13.3p13.1, 9q33.3q34.3, 10p15.3p21.33, 10q25.1q26.3, 11q11q13.5, 11q14.1, 19p13.3p12, 19q12q13.13, 20p11.23p11.21 | 24.04 |
| 25 | P | 63~64,mar1,mar2,inc[cp6] | 9p24.3p13.3 | *12q12, 12q13.11, 12q13.12, 12q13.13, 12q13.2, 12q13.3q14.1, 12q14.1, 12q14.2, 12q14.3, 12q15, 12q21.1, 12q21.2, 12q21.31, 12q21.31q21.32, 12q21.32, 12q21.33, 12q22* | 5p15.33p11, 9q13q34.3, *15q21.3, 15q24.1*, 19p13.3p13.11, 20q11.21q13.33 | 7.20 |
| 26 | R | 41~46,X,-X,dic(1;7)(p36;p11),-2,-4,  +der(5)t(1;5)(p22;p15),del(9)(p13),  +dic (9;?)(p24;?),+12,+16,-19,+mar1,  +mar2[cp7] | 2q23.3q31.1, 2q31.1, 2q31.2, 2q32.1, 2q32.3, 2q33.1, 2q33.2, 2q33.3, 2q34, 2q35, 3q13.12q13.13, 5p15.31, 5p15.31, 5p15.2p13.2, 5p13.1 5p12, 6q15, 6q16.1, 6q16.2, 6q16.3, 6q21, 6q22.1q22.31, 6q25.1q27, 9p24.1p21.1, 15q14, 16p13.3, 18p11.32p11.21 | *12p13.33, 12p13.31, 12p11.23, 12p11.23p11.21, 12q12, 12q13.11, 12q13.12, 12q13.13, 12q13.2, 12q14.1, 12q14.3, 12q15, 12q21.1, 12q21.2, 12q21.31, 12q21.32, 12q21.33, 12q21.33q22, 12q22, 12q23.1, 12q23.2, 12q23.3, 12q24.11* | 1p22.2, 1p22.2p21.3, 1p21.3, 1p13.3p13.2, 2q31.1, 2q31.2, 2q32.1, 2q32.3, 2q33.1, 2q33.2, 2q33.3, 2q34, 2q35, 5q14.3, 5q15, 5q22.1, 5q21.3, 5q22.1, 5q22, 5q22.3q23.1, 5q23.2, 5q31.1, 5q31.2, 5q31.3, 5q32, 5q33.1q33.2, 5q34q35.3, 6q14.1, 6q14.3q15, 6q16.1, 6q16.2, 6q16.3, 6q21, 6q22.1, 6q22.31, 6q22.33, 6q23.2q25.1, 14q11.2, 14q12, 14q13.1, 14q13.2, 14q13.3, 14q21.1, 14q21.2, 14q21.3, 14q22.1, 17q23.3q24.1, 17q24.1q24.2 | 9.86 |
| 27 | R | 66~135,mar1,mar2,inc[cp4]/46,XX[4] | 3p26.3p11.1, 6p25.3p21.1, 9p24.3p21.3, 10, 13, 15, 16p11.2 16q13q24.3, 20q13.31q13.33 | *12p11.22p11.21, 12q12q13.11* | 3q11.2q29, 7p22.3p11.1, 9q12q34.3, 16p13.3p11.2, 16q11.2q13, 17p13.3q12, 20p13q13.31 | 29.22 |
| 28 – 1* | R | 43~48,r1,r2,inc[cp5] | 6p25.3p12.3, 6p12.1, 6q11.1q12, 7q21.3q36.3, 11q13.5q25, 13q11q31.1, 15q13.3q21.3, 22q13.1q13.33 | *12q12, 12q13.11q13.12, 12q13.12, 12q13.13q13.2,12q13.2, 12q13.3,12q13.3, 12q13.3q14.1, 12q14.1, 12q15, 12q21.1, 12q21.2, 12q21.31, 12q21.31q21.32, 12q21.32, 12q21.33, 12q22* | 2p25.3, 2p25.2, 2p24.3, 2p24.2, 2p13.3, 2p12, 2p11.2, 2q22.3, 2q24.3, 2q32.1, 3q26.2, 3q26.32, 3q26.33, 5q22.3, 5q23.1, 6p12.3p12.1, 8q11.23, 8q12.3, 8q13.1, 8q13.2, 8q13.3, 8q21.11, 13q21.1, 18q12.1 | 9.69 |
| 28 – 2* | R | 36~48,XX,+r,+mar,inc[cp6] | 6p25.3p12.3, 6p12.1, 6q11.1q12, 7q21.3q36.3, 13q11q31.1, 15q13.3q21.3, 22q13.1q13.33 | *12q12, 12q13.11q13.12, 12q13.12, 12q13.13q13.2,12q13.2, 12q13.3, 12q13.3q14.1, 12q14.1, 12q15, 12q21.1, 12q21.2, 12q21.31, 12q21.31q21.32, 12q21.32, 12q21.33, 12q22* | 2p25.3, 2p25.2, 2p24.3, 2p24.2, 2p13.3, 2p12, 2p11.2, 2q22.3, 2q24.3, 2q32.1, 3q26.2, 3q26.32, 3q26.33, 5q22.3, 5q23.1, 6p12.3p12.1, 8q11.23, 8q12.3, 8q13.1, 8q13.2, 8q13.3, 8q21.11, 13q21.1, 18q12.1, 19p13.3p13.11 | 8.51 |
| 28 – 3* | R | 41~55,add(4)(q31),add(7)(q11),  add(9)(q11), add(22)(q13),der(?),t(1;?)  (p13;?),r1,r2,mar,inc[cp7] | 6p25.3p12.3, 6p12.1, 6q11.1q12, 7q21.3q36.3, 13q11q31.1, 15q13.3q21.3, 22q13.1q13.33 | *12q12, 12q13.11q13.12, 12q13.13q13.2,12q13.2, 12q14.1, 12q15, 12q21.2, 12q21.31, 12q21.31q21.32, 12q21.32, 12q21.33, 12q22* | 2p25.3, 2p25.2, 2p24.3, 2p24.2, 2p13.3, 2p12, 2p11.2, 2q22.3, 2q24.3, 2q32.1, 3q26.2, 3q26.32, 3q26.33, 5q22.3, 5q23.1, 6p12.3p12.1, 8q11.23, 8q12.3, 8q13.1, 8q13.2, 8q13.3, 8q21.11, 13q21.1, 16q11.2q24.3, 18q12.1, 19p13.3p13.11 | 9.34 |
| 29 - 1 | M | 43~47,XX,add(4)(p14),+r,inc[cp5]/  78~84,idemx2,inc [cp4] | - | *12p13.33, 12q12, 12q13.12, 12q13.13, 12q13.2, 12q13.3, 12q13.3q14.1, 12q14.1, 12q14.2, 12q14.2q14.3, 12q14.3, 12q15, 12q15q21.1, 12q21.1, 12q21.2, 12q21.31, 12q21.22, 12q22, 12q23.1* | 2q12.1q12.2, 2q24.25q13.1, 5q14.1, 5q14.2q14.3, 13q12.3, 13q13.1, 13q13.3, 13q33.2, 13q33.3, 13q3414q11.2, 14q12, 14q13.1, 14q13.2, 14q13.3q21.1 | 0.65 |
| 29 - 2 | R | 46,XY | 7p15.1p11.1, 7q11.21q36.3 | *12p13.33, 12q12, 12q13.12, 12q13.13, 12q13.2, 12q13.3, 12q13.3q14.1, 12q14.1, 12q14.2, 12q14.2q14.3, 12q14.3, 12q15, 12q15q21.1, 12q21.1, 12q21.2, 12q21.31, 12q21.22, 12q22, 12q23.1, 12q23.3* | 2q12.1q12.2, 2q24.25q13.1, 5q14.1, 5q14.2q14.3, 6q24.1q24.2, 6q24.3q25.1, 6q25.2, 6q25.3q26, 7p22.3p15.1, 11p14.2p14.1, 11p12, 11q23.2q24.1, 13q12.3, 13q13.1, 13q13.3, 13q33.2, 13q33.3, 13q34, 14q11.2, 14q12, 14q13.1, 14q13.2, 14q13.3q21.1, 17q24.2q25.3, 21q22.3, 22q13.31 | 7.01 |
| 29 - 3 | M | 45~47,X,der(X)t(X;4)(q22;q12)[2],  del(2)(p21)[2],add(14)(q22)[2],+r[2],  +mar1[2],+mar2[3],inc[cp6]/46,XX[30] | - | *12p13.33, 12q12, 12q13.12, 12q13.13, 12q13.2, 12q13.3, 12q13.3q14.1, 12q14.1, 12q14.2, 12q14.2q14.3, 12q14.3, 12q15, 12q15q21.1, 12q21.1, 12q21.2, 12q21.31, 12q21.22, 12q22, 12q23.1, 12q23.3* | 2q11.1q11.2, 2q12.1q12.2, 2q13q14.1, 2q21.2q21.3, 2q22.1, 2q22.2, 2q24.2, 2q32.1, 2q32.3, 2q33.1, 4, 5q13.1, 5q14.1, 5q14.2q14.3, 6q24.1q24.2, 6q24.3q25.1, 6q25.2, 6q25.3q26, 9, 13q12.3, 13q13.1, 13q13.3, 13q33.2, 13q33.3, 13q3414q11.2, 14q12, 14q13.1, 14q13.2, 14q13.3q21.1, 16q12.1, 17q24.2q25.3 | 11.89 |
| 29 - 4 | M | 30~47,der(7)add(7)(p11)add(7)(q22)[2], mar[4],inc[cp6] | - | *12p13.33, 12q12, 12q13.12, 12q13.13, 12q13.2, 12q13.3, 12q13.3q14.1, 12q14.1, 12q14.2, 12q14.2q14.3, 12q14.3, 12q15, 12q15q21.1, 12q21.1, 12q21.2, 12q21.31, 12q21.22, 12q22, 12q23.1, 12q23.3* | 2q12.1q12.2, 2q24.2, 6q24.1q24.2, 6q24.3q25.1, 6q25.2, 6q25.3q26, 13q12.3, 13q13.1, 13q13.3, 13q33.2, 13q33.3, 13q3414q11.2, 14q12, 14q13.1, 14q13.2, 14q13.3q21.1 | 0.78 |
| 30 - 1 | R | 44~48,r1,r2,mar1,mar2,mar3[3],inc[cp8] | 1p32.3, 1p22.2p21.3, 2q23.3q31.1, 2q31.1, 2q31.11q31.2, 2q31.2, 2q31.3, 2q32.1, 2q32.1q32.2, 2q32.2, 2q32.3, 2q33.1, 2q33.2, 2q33.3, 2q33.3q34, 2q34, 2q35, 2q36.3, 2q37.1, 2q37.2, 2q37.3, 3q13.12q13.13, 5p15.33p15.32, 5p15.32, 5p15.31, 5p15.2, 5p15.1, 5p14.3, 5p14.2, 5p14.1, 5p13.3, 5p13.2, 5p13.1, 5p12, 6q15q16.1, 6q16.1, 6q16.1q16.2, 6q16.3, 6q16.3q21, 6q21, 6q21q22.1, 6q22.1, 6q22.1q22.31, 6q22.31q22.33, 6q23.1, 6q25.1, 11p15.5p13, 15q13.1q13.3, 15q14q22.31, 15q23q26.3, 16p13.3, 17q25.1, 18p11.32p11.21, 20p13p11.21 | *12p13.33m 12p13.31, 12p11.23, 12p11.22, 12p11.22p11.21, 12p11.21, 12q12, 12q13.11, 12q13.12, 12q13.13, 12q13.2, 12q14.1, 12q14.2, 12q14.3, 12q15, 12q21.1, 12q21.2, 12q21.31, 12q21.31q21.32, 12q21.32, 12q21.33, 12q21.33q22, 12q22, 12q23.1, 12q23.2, 12q23.2q23.3, 12q23.3, 12q24.11* | 1p13.3, 1p13.3p13.2, 2q31.1, 2q31.2, 2q31.2q31.3, 2q31.3, 2q31.3q32.1, 2q32.1, 2q32.2, 2q32.2q32.3, 2q32.3, 2q32.3q33.1, 2q33.1, 2q33.1, 2q33.2, 2q33.3, 2q34, 2q34q35, 2q35, 2q35q36.3, 2q36.3, 2q37.1, 2q37.2, 2q37.3, 5q12.3, 5q14.3, 5q15, 5q15q21.1, 5q21.1, 5q21.2, 5q21.3, 5q22.1, 5q22.2, 5q22.3, 5q22.3q23.1, 5q23.1, 5q23.2, 5q31.1, 5q31.2, 5q31.3, 5q32, 5q33.1q33.2, 5q34q35.3 , 6q14.1, 6q15, 6q16.1, 6q16.2, 6q16.2q16.3, 6q16.3, 6q21, 6q22.1, 6q22.31, 6q22.33, 6q23.1q25.1, 9q21.32, 9q21.33, 9q22.2, 9q22.31, 11p13, 11p12, 11p11.2, 13q21.32, 13q21.33, 13q31.3q32.1, 13q32.1, 14q11.2, 14q12, 14q13.1, 14q13.2q13.3, 14q21.1, 14q21.2, 14q21.3, 14q22.1, 15q11.2, 15q12, 15q14, 15q22.31q23, 17q21.31, 17q23.3q24.2 | 13.60 |
| 30 - 2 | R | 40~46,r1,r2,mar1,mar2,inc[cp5] | 2q32.1, 2q32.1q32.2, 2q32.2, 2q32.3, 2q33.1, 2q33.2, 2q33.3, 2q33.3q34, 2q34, 2q35, 2q36.3, 2q37.1, 2q37.2, 2q37.3, 3q13.12q13.13, 5p15.33p15.32, 5p15.32, 5p15.31, 5p15.2, 5p15.1, 5p14.3, 5p14.2, 5p14.1, 5p13.3, 5p13.2, 5p13.1, 5p12, 6q15q16.1, 6q16.1, 6q16.1q16.2, 6q16.3, 6q16.3q21, 6q21, 6q21q22.1, 6q22.1, 6q22.1q22.31, 6q22.31q22.33, 6q23.1, 6q25.1, 15q14, 16p13.3, 16q11.2q24.3, 17q25.1, 18p11.32p11.21 | *12p13.33m 12p13.31, 12p11.23, 12p11.22, 12p11.22p11.21, 12q12, 12q13.11, 12q13.12, 12q13.13, 12q13.2, 12q14.1, 12q14.2, 12q14.3, 12q15, 12q21.1, 12q21.2, 12q21.31, 12q21.31q21.32, 12q21.32, 12q21.33, 12q21.33q22, 12q22, 12q23.1, 12q23.2, 12q23.2q23.3, 12q23.3, 12q24.11* | 2q32.2q32.3, 2q32.3, 2q32.3q33.1, 2q33.1, 2q33.1, 2q33.2, 2q33.3, 2q34, 2q34q35, 2q35, 2q35q36.3, 2q36.3, 2q37.1, 2q37.2, 2q37.3, 5q12.3, 5q14.3, 5q15, 5q15q21.1, 5q21.1, 5q21.2, 5q21.3, 5q22.1, 5q22.2, 5q22.3, 5q22.3q23.1, 5q23.1, 5q23.2, 5q31.1, 5q31.2, 5q31.3, 5q32, 5q33.1q33.2, 5q34q35.3 , 6q14.1, 6q15, 6q16.1, 6q16.2, 6q16.2q16.3, 6q16.3, 6q21, 6q22.1, 6q22.31, 6q22.33, 6q23.1q25.1, 9q21.32, 9q21.33, 9q22.2, 9q22.31, 13q21.32, 13q21.33, 13q31.3q32.1, 13q32.1, 14q11.2, 14q12, 14q13.1, 14q13.2q13.3, 14q21.1, 14q21.2, 14q21.3, 14q22.1, 17q21.31, 17q23.3q24.2 | 10.20 |
| 31 | R | 60~68,XXX,+1,der(3)t(1;3)(p13;q29), der(3)t(3;8)(p11;p11),-4,-6,-8,+9,-10,-11,  add(11)(p11),-12,-14,add(15)(p11),  del(17)(p11),-20,-20,-21,-21,  der(21)t(12;21)(q13;q22)add(12)(q24),  -22,-22,r1,r2[cp8] | 8q11.23q13.1, 8q31.1q21.2, 8q22.1q24.22, 8q24.22q24.3, 10q11.21q26.3, 11p15.5p14.3, 11p14.2p14.3, 11p14.2p14.1, 11p13p12 11p11.2, 14q11.2q32.33, 16p13.3p11.2, 21q11.2, 21q21.1, 21q21.1q21.3, 21q22.11q22.12, 21q22.12q22.22, 21q22.2q22.3, 22q13.31q13.33 | *12p13.1p11.21, 12q12, 12q13.2, 12q13.3q14.1, 12q14.1, 12q14.2, 12q14.2q14.3, 12q15, 12q15q21.1, 12q21.1, 12q21.2, 12q23.3, 12q24.11, 12q24.13, 12q24.31* | 2p16.1p14, 2p12p11.2, 2p11.2, 2q11.1q11.2, 2q11.2q12.1, 2q13, 2q14.1, 2q14.2q14.3, 2q22.2q24.1, 2q33.3q34, 2q35q37.3, 4p16.3p11, 4q12q13.1, 5p13.3p12, 5q31.3, 5q33.3q34, 6p25.3p11.1 6q11.1q13, 6q14.1, 6q23.3, 6q24.3q25.1, 8p11.23p11.21, 8q13.1, 8q21.2, 8q24.22, 9, 10p11.21, 11q21.1, 14q11.2, 15q22.1, 15q22.31q23, 15q25.1, 15q25.3, 15q26.1q26.3, 18q22.1, 18q22.3, 18q22.3q23, 18q23, 20q11.21q13.33 | 24.80 |
| 32 | P | 47,XY,+r[8] | - | *12q13.13, 12q13.2, 12q14.1, 12q14.3, 12q15, 12q24.23* | 18q12.1, 18q12.2, 18q21 | 0.44 |
| 33 | R | 46~48,XY,+add(15)(q11)x2,inc[cp15] | - | *12q13.3q14.1, 12q14.1q14.2, 12q14.3q15, 12q15, 12q21.1, 12q21.2, 12q21.31q21.32, 12q21.32q21.33* | 1q21.1, 1q21.2, 1q21.3, 1q23.2q23.3, 1q24.2q25.2, 5q11.1q35.3 | 5.38 |
| 34 – 1 | R | 84-92,r1,r2,inc[cp5] | 19q12q13.2 | *12q12, 12q13.11, 12q13.13, 12q13.3q14.1, 12q14.1, 12q14.2, 12q14.2q14.3, 12q14.3q15, 12q15, 12q15q21.1, 12q21.1, 12q21.2, 12q21.2q21.32, 12q21.31, 12q21.31q21.32, 12q21.32, 12q21.33, 12q22, 12q23.1, 12q23.2, 12q23.3* | 1p36.13, 1p36.12, 1p35.3, 1p34.3, 1p32.3p32.2, 1p32.1, 1p31.3, 3q22.2, 3q22.2q22.3, 3q22.3, 15q26.3, 19p13.2, 20p12.3, 22q13.2 | 0.92 |
| 34 – 2 | R | 44-47,X,-X,der(2)t(2;13)(p15;q12),  der(5)t(5;18)(p15;q21),-13,-13,  add(15)(p11),-21,-22,r1,r2,r3,inc[cp10]/  84-89,idemx2,inc[cp4] | 15q13.1q13.2, 15q13.3q14, 15q14q15.1, 15q24.2q25.2, 16q11.2q24.3, 21q21.2q22.3 | *12q12, 12q13.11, 12q13.13, 12q13.3q14.1, 12q14.1, 12q14.2, 12q14.2q14.3, 12q14.3q15, 12q15, 12q15q21.1, 12q21.1, 12q21.2, 12q21.2q21.32, 12q21.31, 12q21.31q21.32, 12q21.32, 12q21.33, 12q22, 12q23.1, 12q23.2, 12q23.3* | 13q31.3, 15q25.3, 17p13.3p11.2, 21q11.2q21.1 | 3.72 |
| 35 | P | 46,XX**,t(12;16)(q13;p11)[**3]/  46,idem,+add(6)(q15)[3]/46,XX[3] | 6q13q22.2 | - | - | 1.46 |
| 36 | M | 45~48,XX,+der(1;16)(q10;p10), **t(12;16)(q13;p11),**der(16)t(12;16) (q13;p11),+19,+20[cp6] | - | - | 1q21.1q44, 12q13.3q24.33, 19, 20 | 9.93 |
| 37 | M | 47,XY,del(6)(q13),+8,**t(12;16)(q13;p11)**[10] | 6q12q27 | - | 8 | 7.95 |
| 38 | R | 46,XY | 16q11.2q24.3 | - | 1q21.1q44 | 4.65 |
| 39 – 1 | P | 46,XX,**t(12;16)(q13;p11)**[5] | 15q22.31q23 | - | - | 0.04 |
| 39 – 2 | P | 47,XX,+5,**t(12;16)(q13;p11)**[10] | 15q22.31q23 | - | 5 | 5.73 |
| 41 – 1 | M | 46,XX,del(6)(q21),**t(12;16)(q13;p11)**[5] | 2q31.1q32.2, 6q14.1q22.31 | - | - | 1.55 |
| 41 – 2 | M | 46~47,XX,+add(1)(p13),del(6)(q21), add(8)(p11),**t(12;16)(q13;p11)**,inc**[**cp8] | 6q14.1q22.31 | - | 1q21.1q44 | 4.50 |
| 41 – 3 | R | 46,XX,del(6)(q21),**t(12;16)(q13;p11)**[10] | 2q31.1q32.2, 6q14.1q22.31 | - | - | 1.55 |
| 41 – 4 | M | 46,XX,del(6)(q21),**t(12;16)(q13;p11)**[10] | 2q24.3q37.3, 6q14.1q22.31, 15q26.2 | - | 2q21.1q24.2, 15q26.2q26.3 | 4.88 |
| 42 | P | 46,XY | - | - | - | 0 |
| 43 | R | 47,XX,+8,**t(12;16)(q13;p11)**[7] | - | - | 8 | 4.51 |
| 44 | R | 85~92,XXYY,-7,+8,der(8)t(1;8)  (p13;q12)x2,**t(12;16)(q13;p11)**x2[cp9] | 6 | - | 1q12q44, 8 | 13.34 |
| 46 | P | 46,XY | 15q15.1q22.2 | - | 13 | 3.77 |
| 47 | P | 81-83,del(1)(p13),der(1)t(1;6)  (q32;p21)x2,**t(12;16)(q13;p11)**,  del(16)(q13q22)x2,del(17)(p11)x2,  i(17)(q10),inc[cp5]/46,XY[4] | 16q11.2q24.3 | - | 1q21.1q32.2, 6p25.3p21.2 | 4.76 |
| 48 | P | 46,XY,**t(12;16)(q13;p11)**[3] | - | - | 1q25.1q44 | 2.41 |
| 49 | P | 47,XY,t(4;22)(q31;q11),**t(12;19)(q13;p13)**,  +13[15]/46,XY[6] | - | - | 13 | 3.12 |
| 50 | R | 46,XX,**t(12;16)(q13;p11)**[2]/92,idemx2[8] | 17p13.2p13.1 | - | - | 0.04 |
| 52 | P | 46,XX,add(1)(p22),der(6)t(6;12)(p23;q15),  **t(12;16)(q13;p11)**[17]/46,XX[3] | 15q14q21.2 | - | - | 0.44 |
| 53 | P | 46,XY,del(6)(q13q23),**t(12;16)(q13;p11)**[10] | 6q13q22.31 | - | - | 1.69 |

Abbreviations: P - primary, R - recurrent, M - metastatic; Tumors with asterisks were reclassified as DDLPS according to array-CGH results.

Italics indicates log2 ratio >2, while bold was used to describe the presence of **balanced translocation** t(12;16) in MLPS samples
